# Retraction: SH2 Modified STAT1 Induces HLA-I Expression and Improves IFN-γ Signaling in IFN-α Resistant HCV Replicon Cells

**DOI:** 10.1371/journal.pone.0313111

**Published:** 2024-11-05

**Authors:** The PLOS ONE Editors

After publication of the Expression of Concern [3] on this article [1, 2], additional concerns were raised about results presented in Figs 1 and 11. Specifically:

- In Fig 1B, the blot appears similar to the GADPH and HCV blots in Fig 7 when cropped along the right-hand edge, and when the GADPH and HCV blots in Fig 7 are cropped along the bottom edge.
- In the PKR panel in Fig 11, when contrast is adjusted, the area directly around the bands does not appear to match the overall background of the panel for which no background noise could be detected.

In response to the concerns listed above and in [3], the corresponding author stated that the underlying data for the experiments in question are no longer available.

In light of the extent of the concerns listed above and in [3] that cannot be resolved in the absence of the original underlying data, and which question the reliability of the reported results and conclusions, the *PLOS ONE* Editors retract this article.

SD did not respond to the final editorial decision. BP, SH, PKC, FG, LAB, and XA either did not respond directly or could not be reached.
